# Antenatal magnesium sulfate treatment and risk of necrotizing enterocolitis in preterm infants born at less than 32 weeks of gestation

**DOI:** 10.1038/s41598-020-69785-3

**Published:** 2020-07-30

**Authors:** Ji Young Hong, Jee Youn Hong, Yun-Sun Choi, Yoo-Min Kim, Ji-Hee Sung, Suk-Joo Choi, Soo-young Oh, Cheong-Rae Roh, Hye Seon Kim, Se In Sung, So Yoon Ahn, Yun Sil Chang, Won Soon Park

**Affiliations:** 1 Department of Obstetrics and Gynecology, Samsung Medical Center, Sungkyunkwan University School of Medicine, 81 Irwon-ro, Gangnam-gu, Seoul, 06351 Republic of Korea; 2 Department of Obstetrics and Gynecology, Chung-Ang University Hospital, Chung-Ang University College of Medicine, Seoul, Korea; 3 Department of Pediatrics, Samsung Medical Center, Sungkyunkwan University School of Medicine, Seoul, Korea

**Keywords:** Gastroenterology, Health care

## Abstract

Antenatal magnesium sulfate (MgSO_4_) treatment is widely used for fetal neuroprotection in women at risk of preterm delivery. However, some studies have recently suggested that in utero MgSO_4_ exposure is associated with an increased risk of necrotizing enterocolitis (NEC). This study aimed to investigate the association between antenatal MgSO_4_ treatment and risk of NEC. This retrospective cohort study included 756 infants born at 24‒31 weeks’ gestation. Subjects were classified into three groups: period 1, when MgSO_4_ treatment protocol for fetal neuroprotection was not adopted (n = 267); period 2, when the protocol was adopted (n = 261); and period 3, when the protocol was withdrawn because of concern of risk of NEC (n = 228). Rates of NEC (≥ stage 2b) were analyzed according to time period and exposure to antenatal MgSO_4_. Significant difference in the rate of NEC was not found across the three time periods (2.6% vs. 6.5% vs. 4.8% in periods 1, 2 and 3, respectively, *p* = 0.103). The rate of NEC was comparable between the infants exposed and unexposed to antenatal MgSO_4_ (5.1% vs. 3.6%, *p* = 0.369). These results showed that antenatal MgSO_4_ treatment was not associated with risk of NEC in our study population.

## Introduction

Magnesium sulfate (MgSO_4_) is one of the most commonly used medications in obstetric practice today since its first reported use in 1916^1,2^. Over the past century, MgSO_4_ has been widely used as an anticonvulsant for treating eclampsia, for preventing eclampsia in women with preeclampsia, and as a tocolysis despite controversy regarding its efficacy^3^. Moreover, recent evidence has suggested that antenatal MgSO_4_ may have fetal neuroprotection in women with threatened preterm delivery^4^. Various randomized controlled trials, systematic reviews, and meta-analyses have revealed that MgSO_4_ could lead to about 30–40% relative reduction of moderate to severe cerebral palsy and gross motor dysfunction in surviving infants without adverse perinatal outcome^5–9^. In 2010, the American College of Obstetricians and Gynecologists (ACOG) released a Committee Opinion encouraging MgSO_4_ administration before anticipated early preterm birth less than 32 weeks of gestation to reduce the risk of cerebral palsy^10^.

Some studies have recently raised concerns about gastrointestinal effects of antenatal MgSO_4_ exposure on preterm neonates, including necrotizing enterocolitis (NEC) and spontaneous intestinal perforation (SIP)^11,12^. However, two larger reports by the Pediatrix Medical Group and the Canadian Neonatal Network showed no increase in NEC or SIP in relationship to any antenatal MgSO_4_ exposure^13,14^. Recent meta-analysis and publications also supported these results^9,15,16^. As most evidences regarding association of antenatal MgSO_4_ with gastrointestinal complications have been controversial, the role of antenatal MgSO_4_ in association with gastrointestinal complications is not well established yet^8^.

In our institute, the protocol of antenatal MgSO_4_ treatment for fetal neuroprotection was adopted in 2014. However, concern about the risk of neonatal gastrointestinal complications including NEC and SIP has led to treatment withdrawal after two years. Therefore, the objective of this study was to investigate whether these protocol changes might have any effect on the risk of NEC by comparing outcomes according to time periods classified by the adoption and withdrawal of antenatal MgSO_4_ treatment protocol for fetal neuroprotection. In addition, we investigated whether antenatal MgSO_4_ treatment per se or for fetal neuroprotection was associated with the risk of NEC in preterm neonates at 24‒31 weeks of gestation.

## Results

A total of 756 neonates were enrolled in this study, including 267 (35.3%) during period 1, 261 (34.5%) during period 2, and 228 (30.1%) during period 3. Maternal characteristics including maternal age, parity, and body mass index were similar across the three time periods. However, distributions of singleton, twin, and triplet pregnancies were significantly different among the three time periods (Table 1). Rates of antenatal MgSO_4_ treatment were significantly different across the three time periods (20.9%, 62.1% and 14.9% in periods 1, 2, and 3, respectively, *p* < 0.001). The most common indication of MgSO_4_ treatment during period 2 was fetal neuroprotection whereas that during periods 1 and 3 was severe preeclampsia. A total dose of MgSO_4_ used during period 2 was significantly lower than that during period 1 or period 3 because the period of antenatal MgSO_4_ treatment for fetal neuroprotection was shorter than those used for severe preeclampsia or tocolysis.

**Table 1:** Maternal baseline characteristics and pregnancy outcomes according to time period.

	Period 1 (n = 220)	Period 2 (n = 227)	Period 3 (n = 188)	*P* value
Age (years)	33.3 ± 3.9	33.3 ± 3.9	33.6 ± 3.8	0.751
Body mass index (kg/m^2^)	25.1 ± 4.1	25.1 ± 4.0	25.7 ± 4.0	0.243
Multiparity	90 (40.9)	79 (34.8)	60 (31.9)	0.150
**Plurality**				0.001^1^
Singleton	168 (76.4)	193 (85.0)	150 (79.8)	
Twin	51 (23.2)	34 (15.0)	31 (16.5)	
Triplet	1 (0.5)	0 (0)	7 (3.7)	
Antenatal MgSO_4_ exposure	46 (20.9)	141 (62.1)	28 (14.9)	< 0.001^12^
Dose of MgSO_4_	54.0 ± 62.6	16.4 ± 47.1	63.1 ± 53.1	< 0.001^12^
**Indication of MgSO**_**4**_ **exposure**				< 0.001^12^
Neuroprotection	1 (2.2)	108 (76.6)	0 (0)	
Severe preeclampsia	30 (65.2)	29 (20.6)	25 (89.3)	
Tocolysis	15 (32.6)	4 (2.8)	3 (10.7)	
Antenatal corticosteroids exposure	196 (89.1)	202 (89.0)	179 (95.2)	0.048^123^
**Cycle of corticosteroids**				0.372
Incomplete course	47 (24.0)	37 (18.3)	32 (17.9)	
Complete course	111 (56.6)	132 (65.3)	116 (64.8)	
Rescue course	38 (19.4)	33 (16.3)	31 (17.3)	
**Tocolysis**				
Ritodrine	121 (55.0)	100 (44.1)	91 (48.4)	0.067
Atosiban	85 (38.6)	77 (33.9)	64 (34.0)	0.506
Nifedipine	74 (33.6)	54 (23.8)	39 (20.7)	0.007^3^
**Indication of delivery**				0.268
PTL	97 (44.1)	88 (38.8)	63 (33.5)	
PPROM	64 (29.1)	77 (33.9)	65 (34.6)	
Maternal–fetal indication	59 (26.8)	62 (27.3)	60 (31.9)	
Cesarean delivery	165 (75.0)	166 (73.1)	135 (71.8)	0.763
Histologic chorioamnionitis	102 (46.4)	107 (47.1)	87 (46.3)	0.981

Data are presented as number (percentage) or mean ± standard deviation.

*MgSO*_*4*_ magnesium sulfate, *PTL* preterm labor, *PPROM* preterm premature rupture of membrane.

^1^Significantly different between period 2 and period 3 (Bonferroni test).

^2^Significantly different between period 1 and period 2 (Bonferroni test).

^3^Significantly different between period 1 and period 3 (Bonferroni test).

Neonatal outcomes including gestational age at delivery, birth weight, sex, and rate of 1-min Apgar score < 4 were similar across the three time periods. However, the rate of 5-min Apgar score < 7 was significantly lower during period 3 than that during period 1 (Table 2). NEC (≥ stage 2b) occurred in 41 among 756 neonates (5.4%). There was no significant difference in the rate of NEC (≥ stage 2b) across the three time periods (2.6% vs. 6.5% vs. 4.8% in periods 1, 2, and 3, respectively, *p* = 0.103). The median postnatal age at the onset of NEC was 18.5 days (range, 3–63 days). Early-onset NEC occurred in 14 (40.0%), and late-onset NEC occurred in 21 (60.0%), but there was no significant difference in the age of onset of NEC across the three time periods. Laboratory findings of NEC including metabolic acidosis (pH < 7.2) and thrombocytopenia (platelet < 150,000/μL), and radiologic and sonographic findings of NEC including fixed loop or severe ileus, portal venous gas, pneumoperitoneum/pneumatosis intestinalis, and ascites were similar across the three time periods. Among 35 neonates with NEC (≥ stage 2b), 23 had NEC perforation which was confirmed by pathologic examination after surgery. The most common site of NEC perforation was jejunum, but the locations of NEC perforation were not significant different across the three time periods. The rate of SIP during period 3 was higher than that during period 1.

**Table 2:** Neonatal outcomes according to time period.

	Period 1 (n = 267)	Period 2 (n = 261)	Period 3 (n = 228)	*P* value
GA at delivery (weeks)	28.3 ± 2.5	27.9 ± 2.3	28.3 ± 2.3	0.142
Birth weight (kg)	1.12 ± 0.40	1.07 ± 0.38	1.09 ± 0.38	0.339
Male	145 (54.3)	139 (53.3)	128 (56.1)	0.813
1-min Apgar score < 4	38 (14.2)	44 (16.9)	33 (14.5)	0.656
5-min Apgar score < 7	59 (22.1)	45 (17.2)	30 (13.2)	0.033^1^
Neonatal death	8 (3.0)	14 (5.4)	16 (7.0)	0.119
NEC (≥ stage 2b)	7 (2.6)	17 (6.5)	11 (4.8)	0.103
Stage 2b	2 (28.6)	4 (23.5)	1 (9.1)	0.591
Stage 3a	1 (14.3)	4 (23.5)	1 (9.1)	
Stage 3b	4 (57.1)	9 (52.9)	9 (81.8)	
Onset of NEC (days)	21 (3‒61)	13.5 (3‒53)	21 (3‒63)	0.622
Early-onset	3 (42.9)	8 (47.1)	3 (27.3)	0.571
Late-onset	4 (57.1)	9 (52.9)	8 (72.7)	
Laboratory findings				
Thrombocytopenia	6 (85.7)	13 (76.5)	8 (72.7)	0.812
Acidosis	3 (42.9)	13 (76.5)	9 (81.8)	0.166
Radiology and ultrasound findings				
Fixed loop or severe ileus	7 (100)	17 (100)	11 (100)	> 0.999
Portal venous gas	2 (28.6)	6 (35.3)	3 (27.3)	0.890
Pneumoperitoneum/pneumatosis intestinalis	4 (57.1)	12 (70.6)	4 (36.4)	0.202
Ascites	4 (57.1)	12 (70.6)	8 (72.7)	0.762
NEC perforation	4 (1.5)	10 (3.8)	9 (3.9)	0.188
Ileum	0 (0)	1 (14.3)	0 (0)	0.535
Jejunum	4 (100)	6 (85.7)	8 (88.9)	
Ileum & jejunum	0 (0)	0 (0)	1 (11.1)	
SIP	1 (0.4)	5 (1.9)	10 (4.4)	0.008^1^

Data are presented as number (percentage), mean ± standard deviation or median (range).

*GA* gestational age, *NEC* necrotizing enterocolitis, *SIP* spontaneous intestinal perforation.

^1^Significantly different between period 1 and period 3 (Bonferroni test).

Rates of NEC (≥ stage 2b), NEC perforation, and SIP were not significantly different between the infants exposed and unexposed to antenatal MgSO_4_ (Table 3). And the results were similar in the subgroup analyses stratified by gestational age at delivery (preterm birth less than 26 weeks of gestation and preterm birth at 26‒31 weeks of gestation). Rates of NEC (≥ stage 2b), NEC perforation, and SIP were also not significantly different between the infants exposed and unexposed to antenatal MgSO_4_ for fetal neuroprotection, and the results were similar in the subgroup analyses stratified by gestational age at delivery (Table 4).

**Table 3:** Neonatal outcomes according to exposure to antenatal MgSO_4_ treatment.

		MgSO_4_ unexposed	MgSO_4_ exposed	*P* value	aOR (95% CI)^1^	*P* value
**NEC (≥ stage 2b)**	All infants	26/509 (5.1)	9/247 (3.6)	0.369	0.70 (0.31‒1.55)	0.373
	PTB < 26 weeks	16/122 (13.1)	2/50 (4.0)	0.076	0.27 (0.06‒1.28)	0.100
	PTB 26‒31 weeks	10/387 (2.6)	7/197 (3.6)	0.510	1.19 (0.43‒3.27)	0.734
**NEC perforation**	All infants	18/509 (3.5)	5/247 (2.0)	0.256	0.58 (0.21‒1.64)	0.306
	PTB < 26 weeks	13/122 (10.7)	1/50 (2.0)	0.059	0.18 (0.02‒1.47)	0.109
	PTB 26‒31 weeks	5/387 (1.3)	4/197 (2.0)	0.494	1.32 (0.34‒5.08)	0.691
**SIP**	All infants	9/509 (1.8)	7/247 (2.8)	0.340	1.82 (0.65‒5.10)	0.252
	PTB < 26 weeks	4/122 (3.3)	2/50 (4.0)	1.000	1.13 (0.19‒6.69)	0.894
	PTB 26‒31 weeks	5/387 (1.3)	5/197 (2.5)	0.317	2.35 (0.64‒8.57)	0.196

Data are presented as number (percentage).

*MgSO*_*4*_ magnesium sulfate, *aOR* adjusted odds ratio, *CI* confidence interval, *NEC* necrotizing enterocolitis, *PTB* preterm birth, *SIP* spontaneous intestinal perforation.

^1^Adjusted for plurality, antenatal corticosteroids treatment, nifedipine treatment, and gestational age at delivery.

**Table 4:** Neonatal outcomes according to exposure to antenatal MgSO_4_ treatment for fetal neuroprotection.

		MgSO_4_ unexposed	MgSO_4_ exposed for fetal neuroprotection	*P* value	OR (95% CI)^1^	*P* value
**NEC (≥ stage 2b)**	All infants	26/509 (5.1)	6/126 (4.8)	0.874	0.80 (0.31‒2.03)	0.636
	PTB < 26 weeks	16/122 (13.1)	2/32 (6.3)	0.368	0.44 (0.09‒2.09)	0.299
	PTB 26‒31 weeks	10/387 (2.6)	4/94 (4.3)	0.490	1.41 (0.42‒4.72)	0.576
**NEC perforation**	All infants	18/509 (3.5)	3/126 (2.4)	0.516	0.59 (0.17‒2.06)	0.405
	PTB < 26 weeks	13/122 (10.7)	1/32 (3.1)	0.303	0.29 (0.04‒2.33)	0.242
	PTB 26‒31 weeks	5/387 (1.3)	2/94 (2.1)	0.627	1.41 (0.26‒7.53)	0.690
**SIP**	All infants	9/509 (1.8)	4/126 (3.2)	0.318	1.84 (0.55‒6.20)	0.325
	PTB < 26 weeks	4/122 (3.3)	2/32 (6.3)	0.605	1.88 (0.31‒11.37)	0.492
	PTB 26‒31 weeks	5/387 (1.3)	2/94 (2.1)	0.627	1.59 (0.30‒8.52)	0.586

*MgSO*_*4*_ magnesium sulfate, *aOR* adjusted odds ratio, *CI* confidence interval, *NEC* necrotizing enterocolitis, *PTB* preterm birth, *SIP* spontaneous intestinal perforation.

^1^Adjusted for plurality, antenatal corticosteroids treatment, nifedipine treatment, and gestational age at delivery.

## Discussion

In this study, we investigated the effect of antenatal MgSO_4_ treatment on the risk of NEC by comparing neonatal outcomes before and after two major changes in the antenatal MgSO_4_ treatment protocol (adoption and withdrawal of antenatal MgSO_4_ treatment for fetal neuroprotection). We found that protocol changes had no effect on the rate of NEC in this population. We also found that neither antenatal MgSO_4_ treatment per se or for fetal neuroprotection was associated with the risk of NEC in preterm neonates born at 24‒31 weeks of gestation in this population. These findings are consistent with previous randomized control trials and other recent studies reporting that there is no significant difference in the incidence of NEC between placebo and antenatal MgSO_4_ treatment groups^9,13–20^.

If magnesium causes intestinal injury, the pathogenesis for that injury is unclear. However, the biologic effect of magnesium on intestinal muscle contractility may be a possible mechanism to cause gastrointestinal complications. Magnesium ions can cross the placenta rapidly and its fetal levels increase in proportion with its maternal levels^21,22^. Magnesium ions can replace calcium ions, disrupt actin and myosin interactions, and reduce contractility, thus generating atony of intestine and fecal impaction^23^. They can also increase mesenteric arterial resistance and reduce mesenteric blood flow^24^. The hypomotility of intestine is also due to cholinergic effect of magnesium^24^. Hypomotility may lead to increased water absorption, the formation of stool plugs, and overgrowth of bacteria. An intestinal plug can increase proximal intraluminal pressure which can be particularly detrimental in setting of immature intestine along with decreased blood flow to the intestine of preterm neonates^8^. However, the effect of antenatal MgSO_4_ exposure on development of NEC is not fully understandable, especially in infants who developed late-onset NEC.

Feeding issue, including enteral or parenteral feeding, breast milk or formula feeding, and feeding intolerance, is another important factor for development of NEC in premature infants^25^. In our study, early-onset NEC (within 14 days after birth) occurred in 14/35 (40.0%), and this incidence was similar to that of a large population-based cohort of 16,669 infants with gestational age < 33 weeks of gestation^26^. The development of these early-onset NEC might be associated with failure of or delayed breast milk feeding^27^. Cesarean section, especially emergency cesarean section, is associated with reduced breast milk feeding success or delayed breast milk feeding initiation^28^. In our study, cesarean section rate was over 70% and most of them are performed as emergency surgery. However, although there were no changes in the feeding protocol during the entire study period, we were not able to collect the individual feeding data because this study was a retrospective medical record review. This is one of the main limitations of this study.

The development of NEC is associated with many other contributing factors^25,29^. Among them, the most important risk factor is prematurity. The earlier the gestational age at delivery, the higher the risk of NEC^25^. In our study, the incidences of NEC and SIP in the infants born at less than 26 weeks of gestation were higher than those in the infants born at 26 weeks of gestation or more. Previous studies reporting the association of antenatal MgSO_4_ with NEC and/or SIP have included extremely preterm babies born at less than 25‒26 weeks of gestation^11,12^. Other studies reporting a lack of association between antenatal MgSO_4_ treatment and NEC or SIP have included preterm infants born at ≤ 28‒34 weeks of gestation^13–16^. We included infants born at 24‒31 weeks of gestation, because the indication of antenatal MgSO_4_ treatment protocol for fetal neuroprotection in our institute was those who were at risk of preterm birth at 24‒31 weeks of gestation, and the objective of this study was to investigate whether these protocol changes might have any effect on the risk of NEC in this population. Our results showed that antenatal MgSO4 treatment per se or for fetal neuroprotection was not associated with increased risk of NEC or SIP in babies born at less than 26 weeks of gestation, as well as in babies born at 26‒31 weeks of gestation. However, our sample size was limited to analyze the association between antenatal MgSO_4_ treatment and NEC or SIP in extreme preterm neonates born at less than 26 weeks of gestation. Therefore, a further study is needed to assess the effect of antenatal MgSO_4_ treatment on gastrointestinal complications such as NEC, SIP, and meconium ileus in extreme preterm infants.

The optimal antenatal MgSO_4_ regimen for fetal neuroprotection in terms of dose, maintenance dose, duration, timing, or repeat treatment has not been established yet^30^. Different protocols of antenatal MgSO_4_ treatment might be another plausible cause of conflicting results about the effect of antenatal MgSO_4_ treatment on the risk of NEC. The antenatal MgSO_4_ treatment protocol for fetal neuroprotection in previous studies consisted of loading dose varying from 4 to 6 g intravenous over 20–30 min and maintenance dose varying from none to 1 g or 2 g per hour for 12 h or 24 h^30^. In studies showing adverse neonatal gastrointestinal outcomes in the setting of antenatal MgSO_4_ exposure^11,12^, higher loading dose (6 g) and higher maintenance dose (2 g per hour) were used than those in our study. Total dose of antenatal MgSO_4_ treatment for fetal neuroprotection was higher (50.9 ± 45.7 g or 33.0 ± 19.8 g)^11,12^ than that of our study (6.3 ± 10.3 g). However, studies that used lower dose of MgSO_4_ (4 g of loading dose only or 4 g of loading dose and 1 g per hour of maintenance dose for 24 h), including the Australasian collaborative trial (ACTOMgSO4), European Trial (PREMAG), and our current study, found no relationship between antenatal MgSO_4_ treatment and NEC or SIP^16,18,31^. Therefore, it is necessary to establish optimum regimen for fetal neuroprotection to achieve maximal effectiveness with minimal adverse effects for both fetus and mother, especially for those at risk of preterm birth at less than 26 weeks of gestation.

The strength of our study is that it is a relatively large cohort study examining the association of antenatal MgSO_4_ treatment with risk of NEC in preterm neonates delivered from 24 + 0 weeks to 31 + 6 weeks. Our study investigated neonates with a large sample size of 756 in spite of a single center study like previous two studies^13,14^. Whereas previous studies investigated neonates with a relatively small sample size to demonstrate the association of antenatal MgSO_4_ with NEC and/or SIP^11,12^. However, our study may be underpowered because the sample size was not enough to show a difference in neonatal outcome, especially for the subgroup analyses stratified by gestational age at delivery. Besides, by comparing neonatal outcome before and after two major changes in the antenatal MgSO_4_ treatment protocol, we were able to analyze the effect of antenatal MgSO_4_ treatment on the risk of NEC. However, subjects in the three time period groups were not comparable regarding baseline characteristics and other predictor variables. Therefore, we performed multivariable analyses to adjust for potential confounding factors. In addition, there might be other unidentifiable confounding factors such as feeding issues and practice changes in obstetrics and neonatology over the study period that should have been controlled for the multivariable analysis.

This study was further limited by its inherent disadvantage of a retrospective study design including information bias. However, we tried to minimize information bias by blinding chart reviewers to study question and hypothesis. This study was also limited by inability to determine the total dose, timing, and retreatment of MgSO_4_ administration, especially in women who were transferred from other hospitals.

In conclusion, antenatal MgSO_4_ treatment for fetal neuroprotection was not associated with NEC, mortality, or other short-term neonatal outcomes in this population. A further study is needed to uncover the long-term neurodevelopmental outcome of these babies.

## Methods

This was a retrospective cohort study of neonates delivered from 24 + 0 weeks to 31 + 6 weeks of gestation in Samsung Medical Center, Seoul, Korea from January 2012 to June 2018. Fetal death and major congenital anomalies were excluded. Medical records of mothers and babies were examined independently by obstetricians and neonatologists. Researchers who were blinded to maternal data including antenatal MgSO_4_ treatment reviewed the neonatal data for outcome. This study was approved by the Institutional Review Board for Clinical Research at Samsung Medical Center (IRB No 2019-04-051) and exemption for informed consent was granted because this was a retrospective chart review study. All methods were carried out in accordance with relevant guidelines and regulations.

Subjects were classified into three groups: period 1, from January 2012 to December 2013 when antenatal MgSO_4_ treatment for fetal neuroprotection was not adopted; period 2, from January 2014 to March 2016 when the treatment was routinely used; and period 3, from April 2016 to June 2018 when the treatment was withdrawn because of concern of increased risk of NEC.

Maternal demographic characteristics included maternal age at delivery, body mass index, parity, and plurality. Pregnancy outcomes reported included indications of antenatal MgSO_4_ treatment, type and cycle of antenatal corticosteroids therapy, type of tocolysis treatment, indications for delivery, mode of delivery, and histologic chorioamnionitis. Indications for delivery were classified into preterm labor, preterm premature rupture of membranes, and maternal–fetal indication such as preeclampsia, placenta previa, placenta abruption, and intrauterine growth restriction. Indications of antenatal MgSO_4_ treatment use were fetal neuroprotection, severe preeclampsia, and tocolysis.

MgSO_4_ treatment protocol for fetal neuroprotection consisted of intravenous loading dose of 4 g over 15 to 20 min followed by maintenance infusion of 1 g per hour for 24 h for women having imminent preterm birth within 24 h. Maintenance dose was discontinued if delivery did not occur within 24 h and delivery was no longer considered imminent. It was resumed if the risk of imminent delivery recurred within 6 h. Repeat loading dose and subsequent maintenance therapy were given if risk of imminent delivery recurred after 6 h. MgSO_4_ for treatment of severe preeclampsia and eclampsia was given with the same loading dose followed by a maintenance infusion of 1 g per hour and discontinued at 24 h after delivery. MgSO_4_ was not used for tocolysis in our institute. Women who were given MgSO_4_ for tocolysis in our study population were transferred from other hospitals. Therefore, the exact protocol or dose of MgSO_4_ used in these women was unknown.

Neonatal outcome measures included gestational age at delivery, sex, birth weight, Apgar scores, and neonatal mortality and morbidities. The primary outcome of our analysis was occurrence of NEC (≥ stage 2b). NEC was diagnosed based on modified Bell’s staging criteria^29^. Stage 2b was defined as mild metabolic acidosis (pH < 7.2), mild thrombocytopenia (platelet < 150,000/μL), absent bowel sounds, definite abdominal tenderness with or without abdominal cellulitis or right lower quadrant mass, and portal venous gas with or without ascites. Stage 3a was defined as systemic, intestinal, radiologic signs of stage 2b plus hypotension, bradycardia, disseminated intravascular coagulation, neutropenia, signs of generalized peritonitis, marked tenderness, and distention of abdomen, and definite ascites. Stage 3a was defined as systemic, intestinal, radiologic signs of stage 3a plus pneumoperitoneum. Early-onset NEC was defined as NEC diagnosed within 14 days after birth, and late-onset NEC was defined as NEC diagnosed more than 14 days after birth^26^. Spontaneous intestinal perforation (SIP) was defined as radiological evidence of perforation, a lack of clinical features of NEC, an absence of radiological features of intestinal ischemia (fixed dilated bowel loops, pneumatosis intestinalis), and/or an intra-operative surgical report and/or histopathology assessment indicating a perforation located in the ileum and on the anti-mesenteric border^32^. As a unit policy, minimal enteral feedings (20 mL/kg/day) were initiated within the first 3‒4 h after birth and usually maintained for a week for infants with gestational age of less than 26 weeks. After that, feedings were gradually increased to reach full enteral feeding (150 mL/kg/day) according to the infant's tolerance. For infants with gestational age of 26 weeks or greater, feedings were started within the first 3‒4 h and advanced by 20 mL/kg/day until full enteral feedings. Infants were fed human breast milk; preterm formula milk was given in the absence of mother's own milk. The management of feeding intolerance and the decision of continuing or discontinuing feedings were done by the attending neonatologists. There were no changes in the feeding protocol during the entire study period.

Obtained data were analyzed using the Statistical Package for Social Sciences version 24 (SPSS Statistics; IBM, Armonk, NY, USA). Maternal characteristics, pregnancy, and neonatal outcomes were compared across the three time periods. They were also compared between the infants exposed and unexposed to antenatal MgSO_4_ and between the infants exposed and unexposed to antenatal MgSO_4_ for fetal neuroprotection. Continuous variables were compared using independent-sample parametric (analysis of variance) or nonparametric (Kruskal–Wallis test) tests depending on data normality. Categorical variables were compared using Chi-square test or Fisher's exact test when one or more expected value was less than 5. Bonferroni test was used for post-hoc analysis to correct for multiple comparisons. Multiple logistic regression analysis was performed to evaluate effects of potential confounding variables such as plurality, antenatal corticosteroids treatment, nifedipine treatment, and gestational age at delivery. Results were considered statistically significant when *p* value was less than 0.05. For multiple comparisons, *p* value was adjusted to 0.017 (0.05/3) by Bonferroni correction.

### Ethical approval

This study was approved by the Institutional Review Board for Clinical Research at Samsung Medical Center at April 25, 2019 (IRB No 2019-04-051).

### Conference presentation

This paper was presented at the 39th Annual Meeting of the Society for Maternal-Fetal Medicine, Las Vegas, NV, February 11–16, 2019 (abstract #489).
