# Author Correction: Antenatal magnesium sulfate treatment and risk of necrotizing enterocolitis in preterm infants born at less than 32 weeks of gestation

**DOI:** 10.1038/s41598-020-76319-4

**Published:** 2020-11-16

**Authors:** Ji Young Hong, Jee Youn Hong, Yun-Sun Choi, Yoo-Min Kim, Ji-Hee Sung, Suk-Joo Choi, Soo-young Oh, Cheong-Rae Roh, Hye Seon Kim, Se In Sung, So Yoon Ahn, Yun Sil Chang, Won Soon Park

**Affiliations:** 1 Department of Obstetrics and Gynecology, Samsung Medical Center, Sungkyunkwan University School of Medicine, 81 Irwon‑ro, Gangnam‑gu, Seoul, 06351 Republic of Korea; 2 Department of Obstetrics and Gynecology, Chung‑Ang University Hospital, Chung-Ang University College of Medicine, Seoul, Korea; 3 Department of Pediatrics, Samsung Medical Center, Sungkyunkwan University School of Medicine, Seoul, Korea

Correction to: *Scientific Reports* https://doi.org/10.1038/s41598-020-69785-3, published online 30 July 2020

The original version of this Article contained errors in the Abstract.

“Antenatal magnesium sulfate (MgSO_4_) treatment is widely used for fetal neuroprotection in women at risk of preterm delivery. However, some studies have recently suggested that in utero MgSO_4_ exposure is associated with an increased risk of necrotizing enterocolitis (NEC). This study aimed to investigate the association between antenatal MgSO_4_ treatment and risk of NEC. This retrospective cohort study included 756 infants born at 24‒31 weeks’ gestation. Subjects were classified into three groups: period 1, when MgSO_4_ treatment protocol for fetal neuroprotection was not adopted (n = 267); period 2, when the protocol was adopted (n = 261); and period 3, when the protocol was withdrawn because of concern of risk of NEC (n = 228). Rates of NEC (≥ stage 2b) were analyzed according to time period and exposure to antenatal MgSO_4_. Significant difference in the rate of NEC was not found across the three time periods (2.6% vs. 6.5% vs. 4.8% in periods 1, 2 and 3, respectively, p = 0.103). The rate of NEC was comparable between the infants exposed and unexposed to antenatal MgSO_4_ (5.1% vs. 3.6%, p = 0.369). These results showed that antenatal MgSO_4_ treatment was not associated with risk of NEC in our study population.’’

now reads:

“Antenatal magnesium sulfate (MgSO_4_) treatment is widely used for fetal neuroprotection in women at risk of preterm delivery. However, some studies have recently suggested that in utero MgSO_4_ exposure is associated with an increased risk of necrotizing enterocolitis (NEC). This study aimed to investigate the association between antenatal MgSO_4_ treatment and risk of NEC. This retrospective cohort study included 756 infants born at 24‒31 weeks’ gestation. Subjects were classified into three groups: period 1, when MgSO_4_ treatment protocol for fetal neuroprotection was not adopted (n = 267); period 2, when the protocol was adopted (n = 261); and period 3, when the protocol was withdrawn because of concern of risk of NEC (n = 228). Rates of NEC (≥ stage 2b) were analyzed according to time period and exposure to antenatal MgSO_4_. Significant difference in the rate of NEC was not found across the three time periods (2.6% vs. 6.5% vs. 4.8% in periods 1, 2 and 3, respectively, p = 0.103). The rate of NEC was comparable between the infants unexposed and exposed to antenatal MgSO_4_ (5.1% vs. 3.6%, p = 0.369). These results showed that antenatal MgSO_4_ treatment was not associated with risk of NEC in our study population.’’

These errors have now been corrected in the PDF and HTML versions of the Article.

